# Consumption of Nitrate-Rich Beetroot Juice with or without Vitamin C Supplementation Increases the Excretion of Urinary Nitrate, Nitrite, and *N*-nitroso Compounds in Humans

**DOI:** 10.3390/ijms20092277

**Published:** 2019-05-08

**Authors:** Julia E. Berends, Lauri M.M. van den Berg, Martina A. Guggeis, Nikki F.T. Henckens, Israt J. Hossein, Minke E.J.R. de Joode, Hossy Zamani, Kirsten A.A.J. van Pelt, Nicky A. Beelen, Gunter G. Kuhnle, Theo M.C.M. de Kok, Simone G.J. Van Breda

**Affiliations:** 1 Department of Toxicogenomics, GROW-school for Oncology and Developmental Biology, Maastricht University Medical Center, P.O Box 616, 6200 MD Maastricht, The Netherlands; je.berends@student.maastrichtuniversity.nl (J.E.B.); lauri.vandenberg@student.maastrichtuniversity.nl (L.M.M.v.d.B.); m.guggeis@student.maastrichtuniversity.nl (M.A.G.); n.henckens@student.maastrichtuniversity.nl (N.F.T.H.); i.hossein@student.maastrichtuniversity.nl (I.J.H.); m.dejoode@alumni.maastrichtuniversity.nl (M.E.J.R.d.J.); h.zamani@student.maastrichtuniversity.nl (H.Z.); k.vanpelt@student.maastrichtuniversity.nl (K.A.A.J.v.P.); nicky.beelen@maastrichtuniversity.nl (N.A.B.); t.dekok@maastrichtuniversity.nl (T.M.C.M.d.K.); 2 Department of Food & Nutritional Sciences, School of Chemistry, Food and Pharmacy, The University of Reading, Reading RG6 6AP, UK; g.g.kuhnle@reading.ac.uk

**Keywords:** Beetroot juice, human dietary intervention, nitrate, nitrite, *N*-nitroso compounds, vitamin C

## Abstract

Consumption of nitrate-rich beetroot juice (BRJ) by athletes induces a number of beneficial physiological health effects, which are linked to the formation of nitric oxide (NO) from nitrate. However, following a secondary pathway, NO may also lead to the formation of *N*-nitroso compounds (NOCs), which are known to be carcinogenic in 39 animal species. The extent of the formation of NOCs is modulated by various other dietary factors, such as vitamin C. The present study investigates the endogenous formation of NOCs after BRJ intake and the impact of vitamin C on urinary NOC excretion. In a randomized, controlled trial, 29 healthy recreationally active volunteers ingested BRJ with or without additional vitamin C supplements for one week. A significant increase of urinary apparent total *N*-nitroso Compounds (ATNC) was found after one dose (5 to 47 nmol/mmol: *p* < 0.0001) and a further increase was found after seven consecutive doses of BRJ (104 nmol/mmol: *p* < 0.0001). Vitamin C supplementation inhibited ATNC increase after one dose (16 compared to 72 nmol/mmol, *p* < 0.01), but not after seven daily doses. This is the first study that shows that BRJ supplementation leads to an increase in formation of potentially carcinogenic NOCs. In order to protect athlete’s health, it is therefore important to be cautious with chronic use of BRJ to enhance sports performances.

## 1. Introduction

Consumption of beetroot juice (BRJ) induces a number of beneficial physiological health effects. These positive physiological effects are induced by the nitrate present in BRJ, which contributes to endogenous nitric oxide (NO) formation [1]. NO plays a fundamental role in the regulation of blood flow, muscle contractility, myocyte differentiation, glucose and calcium homeostasis [2]. Physiological effects after consumption of nitrate-rich products such as BRJ include an increased NO bioavailability, a decreased level of oxygen cost during exercise and a reduction of blood pressure (BP) [3,4,5,6]. Therefore, the muscles’ tolerance to high-intensity exercise, efficacy and endurance may be increased by BRJ, which is of relevance for athletes [3]. The interest in the consumption of nitrate-rich BRJ has increased not only amongst sportsmen, but has also gained attention from the scientific field.

**Figure 1:**
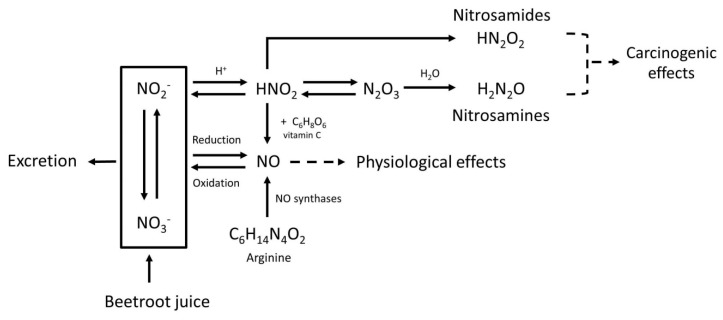
Metabolic pathway of nitrate (NO_3_^−^), nitrite (NO_2_^−^), nitric oxide (NO), nitrosamines, nitrosamides, and the effect of vitamin C. Nitric oxide (NO) is mainly responsible for the physiological effects of beetroot juice (BRJ). The body uses arginine (C_6_H_14_N_4_O_2_) as a source to form NO, and for this reaction oxygen is needed. However, NO can also be formed after intake of nitrate-rich products such as BRJ. Ingested nitrate (NO_3_^−^) will be partly reduced to nitrite (NO_2_^−^) by microflora in the oral cavity. In oxygen-poor environments nitrate and nitrite can be reduced into NO. NO can also be oxidized back into nitrate and nitrite which are water soluble and can therefore be excreted in urine. Under acidic conditions, such as in the human stomach, nitrite will react with the H^+^ and will form HNO_2_ (nitrous acid). Also in the stomach, two molecules HNO_2_ can form N_2_O_3_ (dinitrogen trioxide), by proton catalysis. N_2_O_3_ plays a role in the N-nitrosation rate. Increasing the amount of nitrate will therefore lead to an increase in the N-nitrosation rate. Subsequently, HNO_2_ can react with amides to form nitrosamides (HN_2_O_2_), and N_2_O_3_ can react with amines to form nitrosamines (H_2_N_2_O). Both nitrosamides and nitrosamines are *N*-nitroso compounds and potentially carcinogenic. Vitamin C (C_6_H_8_O_6_) can inhibit the nitrosation process, because it reacts faster than the amine with N_2_O_3_. Vitamin C reduces 2HNO_2_ to NO, and is itself oxidized to dehydroascorbic acid. This will reduce the amount of *N*-nitroso compounds that can be formed.

In addition to beneficial effects, some health risks may also be associated with BRJ. Although the nitrate present in BRJ by itself is non-toxic in humans, the endogenous formation of *N*-nitroso compounds (NOCs) can be stimulated by nitrate derived NO [7]. About 20% of the ingested nitrate will be reduced to nitrite in the oral cavity by bacteria colonizing the saliva. Furthermore, as these bacteria with nitrate reducing activity are also present in other parts of the digestive tract (where pH is above 5), these bacteria will contribute to the endogenous nitrite synthesis, depending on the nitrate available locally. When nitrite is digested in the acidic environment of the stomach, nitrous acid is formed (HNO_2_). Two molecules of HNO_2_ are converted to water and N_2_O_3_, which reacts with amines to nitrosamines. HNO_2_ can also be protonated and can react with amides to form nitrosamides (Figure 1). These NOCs can form reactive intermediates that may bind to DNA causing mutations that are potentially involved in the initiation of carcinogenesis [7].

Human exposure to endogenously formed NOC has been associated with an increased risk of several cancers, such as stomach, esophagus, and bladder cancer [8]. Although no causalities have been established yet in human studies, NOCs have been found to be carcinogenic in at least 39 animal species [9]. These findings suggest that NOCs are likely to be carcinogenic in humans as well [10]. Vermeer et al. (1998) demonstrated that nitrate consumption at the acceptable daily intake (ADI) level (3.7 mg/kg body weight) combined with an amine-rich meal consisting of fish results in an increased NOC excretion in urine in healthy human volunteers [11]. One bottle of concentrated BRJ (70 mL) contains 400 mg of nitrate and thereby exceeding the ADI for most people. As NOCs may be involved in the etiology of human cancers, it is of great relevance to study whether consumption of nitrate-rich BRJ will lead as well to an increased formation and excretion of NOCs in urine. Furthermore, if BRJ consumption will lead to an increase of NOCs, it would be important to investigate a possible way to reduce the formation of NOCs after ingestion of BRJ.

It has been shown that a variety of compounds, including vitamin C, can reduce the formation of NOC by inhibiting the nitrosation reaction [7]. This inhibition occurs in the acidic environment of the stomach when both nitrite and amines are present. Vitamin C reduces HNO_2_ to NO, which is not a nitrosating agent (Figure 1). Furthermore, it reacts faster with N_2_O_3_ than the amines, therefore diminishing the formation of NOCs [7].

In view of the increased interest in BRJ by sportsmen and -women, it is important to investigate its possible health risks [12]. Therefore, we investigated the effect of daily consumption of 70 mL concentrated BRJ containing 400 mg of nitrate in a human dietary intervention study with 29 healthy recreationally active volunteers on the excretion of NOCs and blood pressure. The beetroot juice was given either with or without simultaneous ingestion of 1000 mg vitamin C to establish a potential interfering effect. The excretion of NOCs, nitrate and nitrite in urine was investigated after one day and after seven days of consumption of BRJ. We hypothesize that consumption of 70 mL concentrated BRJ containing 400 mg of nitrate will result in a significant increase in urinary nitrate, nitrite and NOC levels. This effect is expected to be more pronounced after one week of daily consumption, compared to a one-time consumption of BRJ. Furthermore, it is expected that the same amount of BRJ in combination with ingestion of 1000 mg vitamin C will inhibit the excretion of urinary NOC. The results of the study indeed show an increased urinary nitrite, nitrate and NOC level after consumption of BRJ. Vitamin C was only able to inhibit the level of NOC after one dose of BRJ, and not after a longer period of daily BRJ consumption.

## 2. Results

### 2.1. Baseline Characteristics

Twenty-nine subjects completed the study, of which sixteen were female and thirteen were male. One subject dropped-out due to non-compliance to the intervention. No significant differences in baseline characteristics of the subjects were found between the vitamin C group and non-vitamin C group (Table 1).

### 2.2. ATNC Levels in Urine

There was a significant increase in ATNC (*p* < 0.0001) in the participants’ urine after ingestion of BRJ. This was also seen when comparing the ATNC levels on day 1 (5 nmol/mmol) to day 2 (47 nmol/mmol), day 1 to day 8 (104 nmol/mmol), and day 2 to day 8 (Table 2).

**Figure 2:**
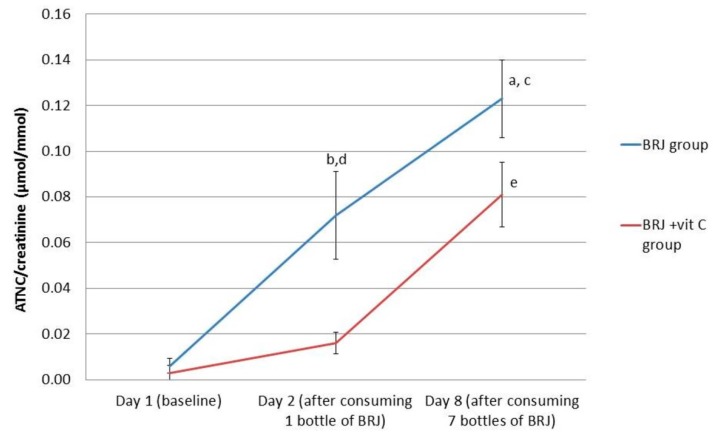
Average values for creatinine corrected ATNC levels in urine (nmol/mmol) for the intervention groups stratified for vitamin C supplementation, at baseline (day 1), after consumption of 1 bottle (day 2) and 7 bottles (1 daily, day 8) of beetroot juice. Error bars indicate standard error. (**a**) *p* < 0.05; significant increase at day 8 compared to day 2 in the BRJ group (Wilcoxon signed rank test); (**b**) *p* < 0.01: significant increase at day 2 compared with baseline levels (day 1) in the BRJ group (Wilcoxon signed rank test); (**c**) *p* < 0.001; significant increase at day 8 compared to baseline levels (day 1) in the BRJ group (Wilcoxon signed rank test); (**d**) *p* < 0.01, significantly higher levels in the BRJ group compared to the BRJ + vit C group (Mann–Whitney test) at day 2; and (**e**) *p* < 0.01, significantly increased levels at day 8 in the BRJ + vit C group compared to baseline levels (day 1) and day 2 (Wilcoxon signed rank test).

The ATNC levels in urine increased after one-time BRJ consumption and increased even further when consuming BRJ for seven consecutive days. After stratification for the use of vitamin C, ATNC levels significantly increased after consumption of BRJ for one week in both groups (*p* < 0.0001 for the BRJ group; *p* < 0.001 for the BRJ + VitC group). For the BRJ group, a one-time consumption of BRJ already resulted in a significant increase of excretion of ATNC in urine (*p* < 0.01). This is in contrast to the BRJ + VitC group, where the increase of ATNC in urine at this time point was not significant (*p* = 0.059) (Figure 2, Table 3).

On day 2, after ingestion of one bottle of BRJ, the ATNC levels in urine of the BRJ + VitC group were significantly lower (16 nmol/mmol) compared to the ATNC levels in urine of the BRJ group (72 nmol/mmol) (*p* < 0.01). However, after consumption of seven bottles of BRJ (day 8), vitamin C had no additional inhibitory effect on ATNC levels in the urine (BRJ group 123 nmol/mmol; BRJ + VitC group 81 nmol/mmol), as ATNC levels were not statistically significantly different between the two groups.

### 2.3. Nitrate Levels in Urine

**Figure 3:**
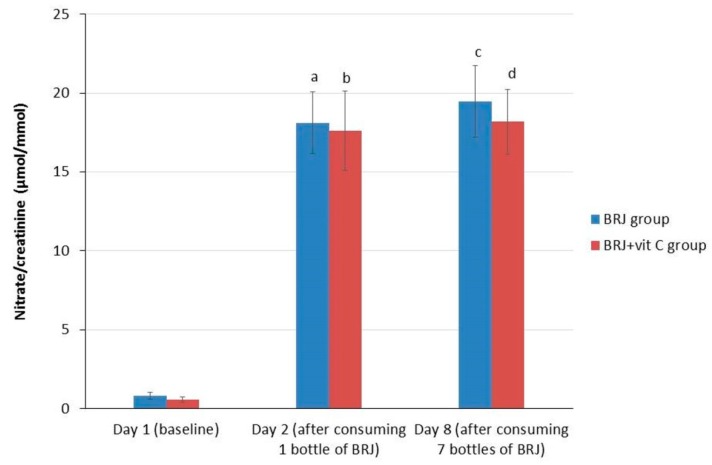
Average values (± SE) for creatinine corrected nitrate levels in urine for the intervention groups stratified for vitamin C supplementation at baseline (day 1), after consumption of 1 bottle (day 2) and 7 bottles (1 daily, day 8) of beetroot juice. Wilcoxon signed rank test; significant increase compared with baseline. (**a**) *p* < 0.001, significant increase at day 2 as compared to baseline levels (day 1) in the BRJ group; (**b**) *p* < 0.001, significant increase at day 2 as compared to baseline levels (day 1) in the BRJ + VitC group; (**c**) *p* < 0.001, significant increase at day 8 as compared to baseline levels (day 1) in the BRJ group; (**d**) *p* < 0.001, significant increase at day 8 as compared to baseline levels (day 1) in the BRJ + VitC group.

BRJ ingestion resulted in a significant increase (*p* < 0.0001) in urinary nitrate in all subjects between day 1 (0.708 µmol/mmol) and day 2 (17.88 µmol/mmol), and between day 1 and 8 (18.89 µmol/mmol; *p* < 0.0001). However, no significant increase between day 2 and day 8 was observed (Table 2). These results showed that a one-time ingestion of BRJ was already sufficient to cause a significant increase of urinary nitrate excretion. Furthermore, nitrate levels in urine remained at the same level after six more days of daily BRJ ingestion. This applied to both the entire group, as for the two separate groups (BRJ group and BRJ + VitC group) when stratified for the use of vitamin C (Table 3, Figure 3).

### 2.4. Nitrite Levels in Urine

After ingestion of one bottle of BRJ (day 2), no significant increase in urinary nitrite was detected. However, after 7 days of daily BRJ consumption (day 8), nitrite concentration in urine increased significantly in the total study population (4 to 21 nmol/mmol; *p* < 0.001), in the BRJ group (2 to 17 nmol/mmol; *p* < 0.05) as well as within the BRJ + VitC group (6 to 25 nmol/mmol; *p* < 0.05) (Table 2, Table 3). There was no significant difference in urinary nitrite levels when comparing the BRJ group with the BRJ + VitC group, neither on day 2 nor day 8.

### 2.5. Vitamin C Levels in Urine

In order to evaluate compliance of vitamin C intake, excretion of vitamin C was determined in urine. After one day of supplementation (day 2), urinary vitamin C concentration in the BRJ + VitC group increased significantly (*p* < 0.01), but did not further increase after an additional six days of vitamin C supplementation (Table 3). No significant increase in urinary vitamin C excretion was measured in the BRJ group at day 2 or day 8. On day 2, the vitamin C levels in urine were not significantly different between the BRJ group and the BRJ + VitC group, but on day 8 this difference was significant (*p* < 0.05).

### 2.6. Blood Pressure

BRJ consumption for seven consecutive days, either with or without vitamin C supplementation, did not result in a significant change in BP (Table 2, Table 3).

## 3. Discussion

Nitrate-rich BRJ is known for its physiologically beneficial health effects due to the formation of NO in the nitrate-nitrite-nitric oxide (NO) pathway. Consumption of nitrate-rich BRJ increases NO bioavailability, decreased oxygen level cost during exercise and blood pressure reduction [3,4,5,6]. Therefore, BRJ may improve muscle endurance, efficacy and tolerance to high-intensity exercise [3]. Other effects of the NO pathway demonstrated by clinical studies are enhancement of blood flow and inhibition of platelet aggregation, therefore possibly protecting BRJ-consumers against cardiovascular disease [13,14]. Additionally, studies have suggested that the NO pathway may be a therapeutic target in patients with pulmonary arterial hypertension [15].

While BRJ is known especially for its beneficial effects on sports performance, it may also have several negative health effects, due to its high nitrate content [8]. Dietary nitrate is known to stimulate the endogenous formation of potentially carcinogenic NOC [16,17]. Several human epidemiological studies have shown that increased formation of NOCs is associated with increased gastrointestinal and bladder cancer risk [8].

The effects of nitrate-rich BRJ on endogenous NOC formation have, to the best of our knowledge, never been investigated in a human intervention study before. Therefore, we recruited 30 healthy participants to evaluate the impact of daily intake of 70 mL of concentrated BRJ containing 400 mg of nitrate on urinary nitrate and nitrite levels and ATNC as marker of endogenous nitrosation [18]. Furthermore, we evaluated the potential impact of vitamin C supplementation on the formation and urinary excretion of ATNC.

Overall, the results showed a significant increase in the excretion of urinary ATNC after consumption of BRJ. This increase was already observed after a single ingestion of 70 mL of BRJ, which significantly increased even further after consumption of one bottle of BRJ/day for six more consecutive days. As a result of urinary excretion no accumulation of Vitamin C occurred. Vitamin C supplementation was able to inhibit ATNC formation after ingestion of the first bottle of BRJ, but this effect was not found after a longer period of the intervention. The excretion of nitrate in urine was significantly elevated after drinking one bottle of BRJ (17.88 µmol/mmol), and remained elevated after six consecutive days of BRJ ingestion (18.89 µmol/mmol). In contrast, the amount of nitrite found in the urine after drinking a single bottle of BRJ was below level of detection for most of the subjects. However, a significant increase in excretion was visible after seven days of BRJ consumption. Finally, no significant effect on blood pressure was observed in this study.

The significant increase of ATNC in urine after consumption of a single bottle of BRJ (400 mg nitrate), as well as the significant increase after six more subsequent days of drinking one bottle of BRJ/day, confirms our hypothesis. ATNC is a marker of endogenous nitrosation and comprises different types of nitroso compounds. Due to the high reactivity of *N*-nitroso compounds and their extensive metabolism, these compounds are a poor marker of overall endogenous nitrosation and are likely to result in an underestimation. ATNC is therefore commonly used as an indicator of endogenous nitrosation [18]. These results show that BRJ intake increased urinary NOCs almost 20-fold. Vermeer et al. (1998) analyzed urinary concentrations of volatile NOC such as *N*-nitrosodimethylamine (NDMA), which are known to be carcinogenic [11]. In healthy participants who consumed a nitrate-rich diet (at acceptable daily intake (ADI) 220 mg/day level), preceded and followed by a nitrate-poor diet, urinary NDMA was measured. This increased from 287 ± 223 ng/24 h in the first nitrate-poor week to a maximum of 871 ± 430 ng/24 h in the nitrate-rich week, and went back to 383 ± 168 ng/24 h in the last nitrate-poor week. This resulted in a significant increase of 2-fold. Therefore, compared to the 2-fold increase in Vermeer’s study, the present study found a much higher increase in urinary ATNC concentration of 20-fold. There are several differences between Vermeer’s study design and the study design of the present study. Firstly, while the present study used morning urine samples to measure ATNC whereas Vermeer used 24 h urine measurements of only NDMA. Secondly, as most other studies investigating the effect of nitrate intake, Vermeer did not use BRJ, but nitrate originating from drinking water. It is remarkable that BRJ, which does potentially contain natural antioxidants that could inhibit nitrosation, apparently has a stronger effect as compared to drinking water nitrate that does not contain any antioxidant capacity. To the best of our knowledge, the effects of nitrate-rich BRJ consumption on ATNC formation and excretion have not been investigated before.

Urinary nitrate excretion increased significantly after a single day of BRJ consumption, but did not result in higher excretion levels of nitrate after additional consumption of BRJ. Instead, these levels remained the same. Another study has also shown a significant increase of urinary nitrate after a single day of nitrate consumption at the ADI level, which is in line with the results of the present study [11]. The lack of a further increase of urinary nitrate after consumption of additional BRJ may be because nitrate is almost completely excreted during the 24 h following ingestion. Previous studies have demonstrated that 75–88% and 65–70% of the ingested nitrate dose was detected in urine within a single day [11,19,20]. The dose of BRJ remained constant in the present study, and nitrate levels in urine also remained constant after day 1.

Potential adverse health effects of high nitrate exposure have been evaluated by the International Agency for Research on Cancer (IARC), and showed associations with different types of cancer risk. For instance, a significant correlation was found between drinking water containing high concentrations of nitrate and the prevalence of gastric cancer [8]. A significantly higher number of people from the town of Worksop (UK), where the drinking water contained 93 mg/L nitrate, had gastric cancer, compared to the control town having 15 mg/L nitrate in drinking water [21,22]. De Roos et al. (2003) found a 2-fold increased risk of colon cancer in participants with nitrate-nitrogen levels greater than 5 mg/L for longer than ten years average, with dietary vitamin C intake below the median (131.8 mg/day) [23]. This suggests that a long-term daily consumption of nitrate-rich BRJ (400 mg in 70 mL) may result in an increased risk of gastric cancer, a hypothesis that needs to be confirmed in independent studies.

Nitrite can be converted into NO and NOCs, however it can also be converted back into nitrate. Nitrite is not detectable in the urine of healthy people, due to the fast oxidation of nitrite into nitrate [11,24]. In contrast, while there is no significant increase of urinary nitrite after day 1, the results of the present study show an increase in urinary nitrite after seven consecutive days of BRJ consumption. Vermeer et al. (1998) discussed nitrate consumption at the ADI level, however, the concentrated BRJ (400 mg in 70 mL) exceeds the ADI level, which is 3.67 mg/kg body weight [25]. The higher nitrate concentration in BRJ may hypothetically result in incomplete conversion of nitrite to nitrate, and thus an increased urinary nitrite excretion after seven days. The World Health Organization (WHO) also reported little urinary nitrite due to nitrate consumption from drinking water [25]. Normally, urinary nitrite is used as a diagnostic value, indicating urinary tract infection. In this study only healthy participants were included. Urinary nitrite as a result of a urinary tract infection is therefore not likely.

Several studies relate NOC formation to different forms of cancer. Several human epidemiological studies have shown that NOC formation is associated with increased gastrointestinal and bladder cancer risk [8]. The European Prospective Investigation into Cancer and Nutrition (EPIC)–Norfolk study investigated the relation between dietary NOCs and cancer in the UK [26]. It was shown that there is a relation between gastrointestinal, especially rectal cancer and dietary NDMA. Keszei et al. (2013) found a potential positive correlation between esophageal squamous cell carcinomas in men and NOC formation, heme iron, and nitrate intake. They also found a positive correlation between increased NDMA intake and gastric non-cardia adenocarcinoma [27]. One cohort study showed a positive relation between NDMA intake and gastric cancer risk in women [28]. While another study did not find an association between NDMA and gastric cancer, they did find an association between NDMA intake and colorectal cancer [29]. Therefore, there is evidence which indicates a correlation between NOC and NDMA intake and cancer risk.

Previous studies have shown that vitamin C supplementation is capable of reducing urinary NOC formation [30,31]. Vitamin C supplementation was only capable of inhibiting a significant increase after consumption of a single bottle of BRJ, as was measured in urine. For the consecutive days, additional vitamin C supplementation might be necessary to reach a similar effect. However, Vermeer et al. (1998) showed no difference in urinary NOC excretion between administration of 250 mg and 1000 mg of vitamin C supplementation [11]. Therefore, it might be unlikely that a higher dose of vitamin C supplementation would inhibit ATNC excretion [7].

Several studies are in alignment with our findings. Mirvish et al. (1996) and Helser et al. (1991) established that proline ingestion with simultaneous vitamin C supplementation resulted in a reduced formation of endogenous NPRO (*N*-nitrosoproline), a non-carcinogenic NOC, after one day [30,31]. However, Vermeer et al. showed that the mean NDMA excretion decreased after a couple days of consumption of a nitrate-rich diet and 1000 mg of vitamin C, which is in contrast to the findings of our study [24]. Our results only show a decreased ATNC after consumption of one bottle of BRJ in combination with vitamin C, but not after seven days of consumption. A notable difference is that Mirvish et al. and Helser et al. focused on NPRO after proline ingestion and that Vermeer et al focused on NDMA specifically, whereas the present study aimed to reveal the presence of ATNC.

Webb et al. (2008) discussed that the consumption of nitrate-rich fruits and vegetables contributes in lowering BP in healthy normotensive men [32]. The results in our study do not support these claims as no significant reduction in BP was found. This could be due to lack of power due to a relatively low number of included participants. Determination of study size was calculated based on expected effects on ATNC levels. To prove the significance of BP reduction, a larger study population might be necessary.

This human intervention study investigated, and demonstrated the formation of NOCs after ingestion of BRJ in healthy subjects. For further research, a larger study population with diverse age categories is recommended. Furthermore, 24-h urine samples or daily spot urine samples could be examined during the testing period to get a more detailed view of the nitrate, nitrite and NOC excretion after consumption of BRJ.

## 4. Materials and Methods

### 4.1. Subjects

Male and female participants were recruited at the Faculty of Health Medicine and Life Science at the University of Maastricht. Thirteen male and sixteen female healthy, recreational sportsmen between the age of 18 and 45 (mean ± SD: age 21 ± 1.7 years, body mass index (BMI) 23.0 ± 2.4, systolic BP 111 ± 11, diastolic BP 65 ± 9) were included in this study (Table 1). “Healthy” was defined as not being diagnosed with a metabolic or cardiovascular disease, as well as not suffering from any mental or psychological conditions. All participants were non-smokers, did not use any medication, did not take vitamin supplementation and were physically active for 1–8 h per week. Furthermore, all participants gave their informed consent for inclusion before they participated in the study. The study was conducted in accordance with the Declaration of Helsinki, and the study protocol was approved by the Medical Ethical Committee of the University Hospital of Maastricht and Maastricht University on 4 April 2016 (METC azM/UM) (METC153054/NL55247.068.15).

### 4.2. Study Design

Subjects were randomized into two groups; one group ingested only BRJ once a day (BRJ group) for seven consecutive days, while the other group ingested BRJ once a day together with one vitamin C tablet containing 1000 mg of vitamin C (Roter, The Netherlands) (BRJ+VitC group), also for seven consecutive days. The BRJ bottles (www.beet-it.com, United Kingdom, Sufford) each held 70 mL of BRJ containing 400 mg of nitrate.

**Figure 4:**
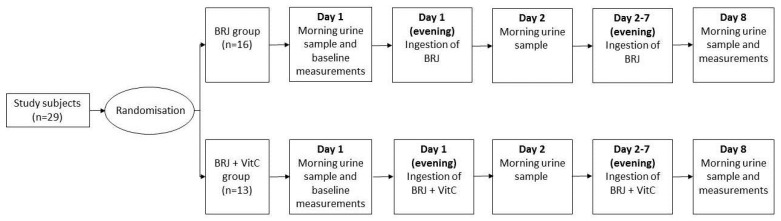
Outline of the experimental protocol. The 29 study subjects were first randomized by block randomization. Sixteen subjects were attributed to the BRJ group and 13 to the BRJ + VitC group. On day 1, the subjects collected morning urine, and the baseline measurements (resting blood pressure and body mass index) were performed. In the evenings of days 1 to 7, the BRJ group ingested only beetroot juice (BRJ), while the BRJ + VitC group ingested both BRJ and vitamin C (BRJ+VitC). On day 2 and 8, all subject collected a morning urine sample. On day 8, the resting blood pressure and body mass index were measured again.

On day 1, baseline measurements (a morning urine sample, BP, and Body Mass Index (BMI)) were taken. The morning urine sample was collected in a 50 mL Greiner tube by the subjects and was immediately stored at −20 °C. At the university, the subjects’ BMI was recorded and the systolic and diastolic BP was measured after a five-minute resting period. The BP was taken three times in a sitting position, alternating arms after each measurement, using a Welch Allyn 767 Desk Aneroid blood pressure monitors with an adult cuff (Daxtrio, The Netherlands, Zaandam). All subjects received seven bottles of BRJ and the BRJ+VitC group also received seven vitamin C tablets. The subjects were instructed to drink one bottle of BRJ (with/without vitamin C) in the evening of day 1 to day 7. In the morning of day 2 and day 8, the subjects collected their urine and stored the samples in a freezer at −20 °C. On day 8, the subjects were asked to bring the two urine samples to the laboratory, and their BP and BMI were measured again (Figure 4).

### 4.3. Analyses of Nitrate and Nitrite in Urine

To analyze nitrate and nitrite concentrations in urine, chemiluminescence was used [33]. The urine samples and standards containing nitrate were initially reduced to NO. After this, they were quantified with an NO analyzer (Nitric Oxide Analyzer (NOA 280i) from SIEVERS, Boldon, UK). Next, the samples were added to 0.05 mol/L vanadium (III)chloride in 1 M hydrochloric acid, refluxing at 90 °C. The NaNO_2_ standards were injected with disposable plastic syringes and needles. This was done in dilutions of 1:10 or 1:20 in triplicates. After this, the samples were mixed using helium gas (purity 99.996%) and the released NO was transferred to the detector. EDAQ Software showed concentrations as nitrate equivalent concentrations. The calibration of the system was done at the beginning of each batch with at least five different concentrations between 1.22–19.5 µM of NaNO_2_.

### 4.4. Analysis of Vitamin C in Urine

To determine the compliance of the subjects in the BRJ+VitC group regarding vitamin C intake, and to compare the vitamin C levels in urine of both study groups, the vitamin C concentration in the urine samples was determined with the indophenol method using spectrometry [34,35,36]. Urine was centrifuged and the supernatant was kept for analysis. A 0.1 M sodium citrate buffer (pH 3.5) and 50 µM 2.6-dichlorophenol indophenol DCPIP solution were prepared. A calibration curve was prepared using a serial dilution of 100 mg/100 mL vitamin C (standard solution). Next, 250 µL of the standard solution or urine sample, 0.5 mL 0.1 M citrate buffer, and 250 µL DCPIP solutions were mixed together. In the presence of vitamin C, the blue colored DCPIP is reduced to the colorless compound dichlorophenolindophenol (DCPIPH_2_). The absorbance of DCPIP is measured at 520 nm using a spectrophotometer.

### 4.5. Analyses of Creatinine Levels in Urine

As morning urine samples were collected instead of 24 h urine, creatinine was measured in urine in order to correct for urine volume. The creatinine concentration in the urine samples was determined via an ILAB-600 clinical analyzer (Warrington, UK) using the Jaffe method [37]. In short, urine samples were centrifuged at 2500 RPM for 5 min before analysis. Next150 uL of the urine samples, quality controls, and a series of dilutions of a stock creatinine solution for creating a standard curve, were pipetted into 3 mL containers. These were inserted in the ILAB-600 analyzer together with 0.75 N NaOH and 0.04 N picric acid. The intensity of the red color at 520 nm was measured against the blank, as measure of creatinine.

### 4.6. Analysis of Apparent Total Nitroso Compounds (ATNC) in Urine

Apparent total nitroso compounds (ATNC) were used as surrogate marker of total nitroso compounds in urine. Urine samples were analyzed for ATNC by means of chemiluminescence (see above) [33]. For the analysis, 100 μL of the urine sample was treated with preservation solution (0.1 M *N*-ethylmaleimide and 0.01 M DTPA), followed by incubation with 50 g/L sulfamic acid for 1–5 min to remove nitrite. Afterwards, the sample was injected to the purge vessel (60 °C) which contained 10–15 mL reduction solution (140 mL glacial acetic acid, 5.55 g/L iodine in 40 mL water and 11.11 g/L potassium iodide). To avoid a change in the nitrosation state of thiols, preservation solution was added. The mechanism through which this is achieved is based on the alkylation of free thiol groups and scavenging metal ions, which can trigger a release of NO from nitroso-thiols. Tri-iodide reduction solution (kept at 4 °C) released NO from nitrosothiols, nitrosamines, and nitrosyl-hemoglobin.

### 4.7. Statistical Analysis

Statistical analysis of the data was carried out using SPSS 21.0 (IBM, United States). A *p*-value < 0.05 was considered to be statistically significant. Per protocol analysis was performed.

As morning urine samples were collected instead of 24 h urine, levels of ATNC, nitrate, nitrite, and vitamin C in urine were first corrected for urinary volume by means of urinary creatinine levels. For the four analyzed parameters (creatinine-corrected concentrations of ATNC, nitrate, nitrite, and vitamin C in urine), normality of data was tested with the Shapiro–Wilk test (sample size <50). As data was not normally distributed, non-parametric tests were used for all subsequent comparisons.

With the Friedmann test, the overall change in the four parameters between the different test days was analyzed. The Wilcoxon signed rank test was then used to determine the difference between day 1 and 2, and day 1 and 8. Both tests were repeated to analyze differences for the whole study population as well as for the two stratified groups (BRJ group and BRJ + VitC group). The difference in the parameters between day 1 and 2, and day 1 and 8 between the BRJ and BJR + VitC group was subsequently analyzed with the Mann–Whitney test.

BP data were normally distributed, as tested with the Shapiro-Wilk test. Paired t-test was used to analyze differences in systolic or diastolic BP between day 1 and day 8. The independent t-test was performed to compare the difference in BP between the BRJ group and BRJ + VitC group on day 1 and 8.

## 5. Conclusions

As hypothesized, daily consumption of 70 mL concentrated BRJ containing 400 mg of nitrate after one day and one week, results in a significant increase in urinary NOC levels (measured as ATNC). Vitamin C supplementation was able to inhibit NOC excretion after ingestion of only one bottle of BRJ, but not for a longer period. A significant increase of nitrite is found after consumption of seven days of BRJ. In contrast, the excretion of nitrate in the urine is significantly elevated after one day of drinking one bottle of BRJ, which remains constant after six consecutive days of BRJ consumption. This is the first study demonstrating an increased formation and excretion of possible carcinogenic NOCs after ingestion of high-nitrate levels through BRJ consumption. In order to protect the health of sportsmen, it is therefore important to be cautious with chronic use of BRJ to enhance sports performances and more research is needed to exclude possible long-term adverse health effects.

## Figures and Tables

**Table 1:** Baseline characteristics of the study subjects.

	All Subjects (*n* = 29)	BRJ + Vit C Group (*n* = 13)	BRJ Group (*n* = 16)
Females/males	16/13	9/4	7/9
Age (y)	21 ± 0.3	21 ± 0.6	21 ± 0.3
BMI day 1 (kg/m^2^)	23.0 ± 0.4	22.9 ± 0.6	23.2 ± 2.7
BMI day 8 (kg/m^2^)	23.0 ± 0.5	23.0 ± 0.5	23.0 ± 0.7

Data in mean ± SE. BMI: Body Mass Index.

**Table 2:** Blood pressure (BP), creatinine corrected apparent total *N*-nitroso compounds (ATNC), nitrate, nitrite and vitamin C levels in urine at baseline, after consumption of 1 bottle (day 2) and 7 bottles (1 daily, day 8) of beetroot juice for all subjects.

	All Subjects (*n* = 29) ^1^			*p*-Values ^2^		
	Day 1 (baseline)	Day 2	Day 8	Day 1–2	Day 1–8	Day 2–8
Systolic BP (mmHg)	111 (106–115)	NA	112 (108–116)	NA ^3^	NS ^3^	NA ^3^
Diastolic BP (mmHg)	65 (62–68)	NA	63 (62–68)	NA ^3^	NS ^3^	NA ^3^
ATNC/creatinine (nmol/mmol)	5 (<1; 9)	47 (23; 71)	104 (0.080; 0.128)	<0.0001	<0.0001	<0.01
Nitrate/creatinine (µmol/mmol)	0.708 (0.416; 1000)	17.88 (14.75; 21.01)	18.89 (15.75; 22.04)	<0.0001	<0.001	NS
Nitrite/creatinine (nmol/mmol)	4 (0; 9)	8 (0; 24)	21 (12; 29)	NS	<0.001	<0.001
Vitamin C/creatinine (mmol/mmol)	0.35 (0.23; 0.47)	0.59 (0.46; 0.71)	0.50 (0.39; 0.60)	<0.001	<0.05	NS

NA: Not applicable; NS: Not significant; ^1^ Values are in mean (95% Cis); ^2^ Wilcoxon signed rank test unless otherwise stated; ^3^ based on paired sample *T*-test.

**Table 3:** Blood pressure (BP), creatinine corrected apparent total *N*-nitroso compounds (ATNC), nitrate, nitrite, and vitamin C levels in urine at baseline, after consumption of 1 bottle (day 2) and 7 bottles (1 daily, day 8) of beetroot juice for the groups stratified for vitamin C supplementation.

	BRJ + Vit C Group (n = 13) ^1^			*p*-Values *^1^			BRJ Group (*n* = 16) ^1^			*p*-Values ^2^			*p*-Values Groups Compared ^3^	
	Day 1 (baseline)	Day 2	Day 8	Day 1–2	Day 1–8	Day 2–8	Day 1 (baseline)	Day 2	Day 8	Day 1–2	Day 1–8	Day 2–8	Day 2	Day 8
SBP (mmHg) ^1^*^3^	107(101; 114)	NA ^2^	109 (102; 116)	NA	NS ^3^	NA	113 (107; 119)	N/A	114 (108;119)	NA ^4^	NS ^4^	NA ^4^	NA ^5^	NS ^5^
DBP (mmHg) *^3^	64 (58; 70)	NA	62 (58; 66)	NA	NS	NA	66 (61; 70)	N/A	65 (60; 70)	NA ^4^	NS ^4^	NA ^4^	NA ^5^	NS ^5^
ATNC/creatinine (nmol/mmol)	3 (0; 10)	16 (6; 27)	81 (50; 112)	NS	<0.01	<0.01	6 (0; 13)	72 (31; 113)	123 (86; 159)	<0.01	<0.0001	<0.05	<0.01	NS
Nitrate/creatinine (µmol/mmol)	0.56 (0.17; 0.95)	17.61 (12.13; 23.09)	18.19 (13.71; 22.66)	<0.001	<0.001	NS	0.82 (0.37; 1.28)	18.11 (13.96; 22.25)	19.47 (14.61; 24.32)	<0.0001	<0.0001	NS	NS	NS
Nitrite/creatinine (nmol/mmol)	6 (0; 17)	0 (0; 0)	25 (11; 39)	NS	<0.05	<0.01	2 (0; 4)	15 (14; 44)	17 (5; 30)	NS	<0.05	<0.05	NS	NS
Vitamin C/creatinine (mmol/mmol)	0.42 (0.17; 0.68)	0.78 (0.56; 0.99)	0.68 (0.52; 0.84)	<0.01	NS	NS	0.29 (0.20; 0.39)	0.43 (0.30;0.56)	0.35 (0.24; 0.46)	NS	NS	NS	NS	<0.05

NA: not applicable; NS: not significant; SBP: systolic blood pressure; DBP: diastolic blood pressure; ^1^ Values are in x; 95% Cis; ^2^ Wilcoxon signed rank test; ^3^ Mann–Whitney U test; ^4^ independent *T*-test; ^5^ paired sample *T*-test.

## Abbreviations

BRJ	beetroot juice
NO	nitric oxide
BP	blood pressure
NOC	*N*-nitroso compound
ADI	acceptable daily intake
ATNC	apparent total *N*-nitroso compounds
CLD	chemiluminescence detector
NDMA	*N*-nitrosodimethylamine
